# Supplementary material for: The dynamic nature of the human origin recognition complex revealed through five cryoEM structures
Source: eLife. 2020 Aug 18;9:e58622. doi: 10.7554/eLife.58622 (PMC7467728; doi:10.7554/eLife.58622)
Supplement: Supplementary file 1. [file elife-58622-supp1.docx]

**Supplement Table 1 – CryoEM and model statistics of all ORC structures.**

| **Data Collection** | ORC_O1AAA_ | ORC_O2WH_ | ORC_OPEN_ | ORC_DNA_ | ORC_O2-5_ |  |  |  |
| --- | --- | --- | --- | --- | --- | --- | --- | --- |
| Microscope | Titan Krios | Titan Krios |  |  |  |  |  |  |
| Voltage (kV) | 300 | | | | 300 |  |  |  |
| Nominal magnification | 130,000 | | | | 130,000 |  |  |  |
| Exposure navigation | Stage Position | | | | Stage Position |  |  |  |
| Cumulative exposure (e^-^ Å^-2^) | 66 | | | | 77 |  |  |  |
| Exposure rate (e^-^ Å^-2^ sec^-1^) | 11 | | | | 11 |  |  |  |
| Electrons per frame (e^-^ Å^-2^) | 2.2 | | | | 2.2 |  |  |  |
| Detector | K2 Summit | | | | K2 Summit |  |  |  |
| Pixel size (Å) | 1.07 | | | | 1.07 |  |  |  |
| Defocus range (µm) | -1.0 to -2.4 | | | | -1.0 to -2.4 |  |  |  |
| Micrograph used | 9,068 | | | | 4,627 |  |  |  |
| Total extracted particles (no.) | 2,097,508 | | | | 494,812 |  |  |  |
| Refined particles (no.) | 854,972 | | | | 209,313 |  |  |  |
|  |  | | | |  |  |  |  |
| **Reconstruction** |  |  |  |  |  |  |  |  |
| Final particles (no.) | 160,500 | 60,001 | 83,895 | 37,734 | 53,009 |  |  |  |
| Symmetry imposed | C1 | C1 |  |  |  |  |  |  |
| Map sharpening B-factor (Å^2^) | -45 | | | | -68 | -160 | -231 | -66 |
| Resolution (half maps) |  |  |  |  |  |  |  |  |
| FSC 0.143 (unmasked/masked) | 3.4/3.2 | 3.8/3.6 | 4.3/4.0 | 4.7/4.4 | 3.6/3.5 |  |  |  |
| Resolution (models) |  |  |  |  |  |  |  |  |
| FSC 0.5 (unmasked/masked) | 3.6/3.4 | 3.9/3.8 | 7.1/4.5 | 8.3/7.9 | 3.7/3.6 |  |  |  |
| FSC 0.143 (unmasked/masked) | 3.1/3.1 | 3.6/3.5 | 4.1/3.8 | 4.3/4.1 | 3.5/3.4 |  |  |  |
|  |  |  |  |  |  |  |  |  |
| **Atomic Models** |  |  |  |  |  |  |  |  |
| Protein residues | 1966 | 1816 | 2045 | 1932 | 1691 |  |  |  |
| Map correlation coefficient | 0.77 | 0.78 | 0.65 | 0.56 | 0.82 |  |  |  |
| R.M.S. Deviation |  |  |  |  |  |  |  |  |
| Bond lengths (Å) | 0.007 | 0.007 | 0.007 | 0.006 | 0.008 |  |  |  |
| Bond angles (°) | 1.289 | 1.144 | 1.366 | 1.144 | 1.094 |  |  |  |
| Ramachadran |  |  |  |  |  |  |  |  |
| Outliers (%) | 0.00 | 0.00 | 0.00 | 0.00 | 0.00 |  |  |  |
| Allowed (%) | 3.01 | 1.57 | 3.59 | 3.12 | 3.02 |  |  |  |
| Favored (%) | 96.99 | 98.43 | 96.41 | 96.88 | 96.98 |  |  |  |
| Poor rotamers (%) | 0.00 | 0.00 | 0.00 | 0.00 | 0.00 |  |  |  |
| MolProbity Score | 1.19 | 1.11 | 1.53 | 1.39 | 1.17 |  |  |  |
| Clashscore (all atoms) | 2.36 | 3.22 | 5.37 | 4.24 | 2.16 |  |  |  |
| CaBlam Score (%) | 1.30 | 1.48 | 1.73 | 1.57 | 2.23 |  |  |  |
|  |  |  |  |  |  |  |  |  |
